# Factors associated with hepatitis C antibody seroconversion after transplantation of kidneys from hepatitis C infected donors to hepatitis C naïve recipients

**DOI:** 10.1080/0886022X.2020.1798784

**Published:** 2020-07-30

**Authors:** Uchenna Agbim, Orsolya Cseprekal, Masahiko Yazawa, Manish Talwar, Vasanthi Balaraman, Anshul Bhalla, Pradeep S. B. Podila, Benedict Maliakkal, Satheesh Nair, James D. Eason, Miklos Z. Molnar

**Affiliations:** a Department of Surgery, University of Tennessee Health Science Center, Memphis, TN, USA; b James D. Eason Transplant Institute, Methodist University Hospital, Memphis, TN, USA; c Department of Transplantation and Surgery, Semmelweis University, Budapest, Hungary; d Division of Nephrology and Hypertension, St. Marianna University School of Medicine, Tokyo, Japan; e Faith and Health Division, Methodist Le Bonheur Healthcare, Memphis, TN, USA; f Division of Health Systems Management and Policy, School of Public Health, The University of Memphis, Memphis, TN, USA

**Keywords:** Seroconversion, hepatitis C virus, transplantation, policy

## Abstract

**Background:**

We aimed to assess the probability and factors associated with the presence of hepatitis C virus (HCV) antibody among HCV seronegative kidney transplant recipients receiving HCV-infected (nucleic acid testing positive) donor kidneys.

**Methods:**

This is a retrospective review examining HCV antibody seroconversion of all kidney transplant recipients receiving an organ from an HCV-infected donor between 1 March 2018 and 2 December 2019 at a high-volume kidney transplant center in the southeast United States.

**Results:**

Of 97 patients receiving HCV-infected kidneys, the final cohort consisted of 85 recipients with 5 (5.9%) recipients noted to have HCV antibody seroconversion in the setting of HCV viremia. The HCV RNA level at closest time of antibody measurement was higher in the seroconverted patients versus the ones who never converted [median and (interquartile range): 1,091,500 (345,000–8,360,000) vs 71,500 (73–313,000), *p* = 0.02]. No other significant differences including type of immunosuppression were noted between the HCV antibody positive group and HCV antibody negative group. Donor donation after cardiac death status [Odds Ratio (OR) and 95% Confidence Interval (CI) was: 8.22 (1.14–59.14)], donor age [OR (95% CI) (+5 years) was: 3.19 (1.39–7.29)] and Kidney Donor Profile Index [OR (95% CI) (+1) was:1.07 (1.01–1.15)] showed a statistically significant association with HCV seroconversion.

**Conclusions:**

HCV antibody should not be considered routine screening for presence of infection in previously HCV naïve kidney transplant recipients receiving kidneys from HCV-infected donors, as only a modest percentage have antibody despite active viremia. The assessment of HCV viral load should be routine in all transplant recipients receiving organs from public health service increased risk donors.

## Introduction

Hepatitis C virus (HCV) is an infection causing hepatic and extrahepatic complications affecting over 2 million people in the United States [1]. Numerous groups and societies including the Centers for Disease Control and Prevention (CDC), American Association for the Study of Liver Diseases (AASLD), and the Infectious Diseases Society of America (IDSA) recommend screening for the virus using HCV antibody followed by HCV ribonucleic acid (RNA) if the former is positive [2,3]. More specifically, recommendations call for assessing HCV status with HCV RNA in transplant recipients [4], but HCV antibody alone is often checked instead [5], despite guidance in this particular group. However, HCV antibody remains positive independent of virologic cure or ongoing infection. Current HCV third-generation enzyme-linked immunosorbent assays have intermediate sensitivity ranging from 61 to 81.8%, but excellent specificity of 97.5–99.7% for detection of HCV for detection of HCV in the general population [6].

Traditionally, priority for organs from HCV-infected [nucleic acid testing (NAT) positive)] donors was given to HCV-infected recipients as treatment options for HCV were limited due to poor efficacy and intolerable side effects. However, the revolutionary direct-acting antiviral agents (DAAs) has markedly transformed the HCV treatment landscape with virologic cure rates approaching 99%, such that transplanting HCV-infected organs into HCV naïve recipients is now a reality [7–9]. In parallel with the life-saving HCV therapy, increasing rates of unintentional overdoses from the opioid epidemic resulting in deaths have contributed to a considerable number of organs from otherwise relatively young, healthy donors [10] also referred to as public health service (PHS) increased risk organ (IRO) meaning organs at risk for human immunodeficiency virus (HIV), hepatitis B virus (HBV), or HCV [4]. Evidence demonstrates HCV-infected organ donation to HCV naïve recipients in non-hepatic organs can be performed under stringent protocolized settings [8,9] as well as in real-world settings [7]. At present, the majority of studies have focused on feasibility and safety of infected organ donation to HCV naïve recipients [7–9,11]. While a few studies have evaluated the natural history of HCV seroconversion in seronegative organ transplant recipients receiving HCV-infected organs [12] or HCV antibody positive/NAT negative organs [13], the factors associated with HCV antibody seroconversion have not been fully elucidated in this population. The aim of this study was to report natural history and determinants associated with HCV antibody seroconversion in HCV naïve kidney transplant recipients upon the receipt of HCV-infected kidneys.

## Materials and methods

### Cohort definition and data source

**Figure 1.**
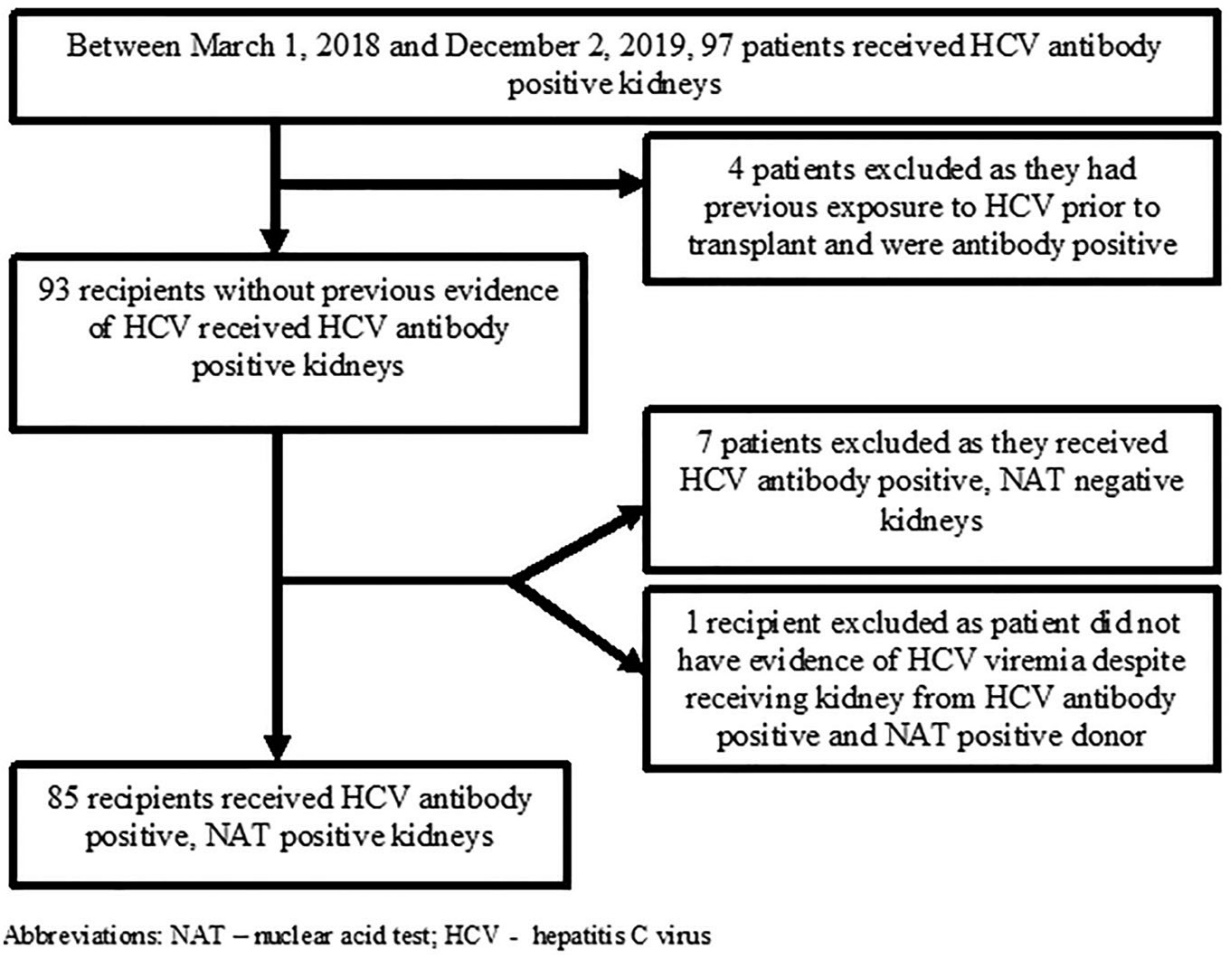
Flow chart of selection of the patients. NAT: nuclear acid test; HCV: hepatitis C virus

The study population included all kidney transplant recipients receiving an HCV antibody positive and/or NAT positive kidney between 1 March 2018 and 2 December 2019 at our center. Recipients who had evidence of previous HCV-infection as indicated by a positive HCV antibody prior to or at the time of transplant were excluded (Figure 1). Furthermore, recipients receiving HCV antibody positive, NAT negative kidneys were also excluded (Figure 1).

All of the data were extracted from our local transplant dataset, hospital electronic medical record system, and the United Network for Organ Sharing system. All study data were collected, managed, and stored in the Research Electronic Data Capture electronic data capture tool hosted by the University of Tennessee Health Science Center [14]. REDCap (Research Electronic Data Capture) is a secure, web-based application designed to support data capture for research studies, providing: 1) an intuitive interface for validated data entry; 2) audit trails for tracking data manipulation and export procedures; 3) automated export procedures for seamless data downloads to common statistical packages; and 4) procedures for importing data from external sources. Data included recipient and donor demographic information (age, race, sex), recipient and donor comorbidities, and laboratory values such as HCV RNA, aspartate aminotransferase (AST), alanine aminotransferase (ALT), creatinine, estimated glomerular filtration rate (eGFR) as well as transplantation related data such as cold ischemic time, induction type and dose and Human Leukocyte Antigen (HLA) mismatches.

### Induction and maintenance immunosuppression protocol

All recipients received rabbit anti-thymocyte globulin (ATG) induction therapy with a planned cumulative dose of 4.5 mg/kg divided into three doses. All recipients were started on a triple immunosuppressive regimen consisting of tacrolimus, mycophenolic acid, and prednisone unless they had a contraindication, and remained on a maintenance dose of prednisone 5 mg daily per protocol.

### Outcome assessment

The primary outcome of this study was HCV antibody seroconversion. HCV antibody was assessed on demand at a physician’s discretion and detected as reactive or non-reactive (positive or negative, respectively) using chemiluminescence run on the Siemens ADVIA Centaur® XPT Immunoassay System per the manufacturer’s instructions in the immunology laboratory at Methodist University Hospital in Memphis, TN. The HCV antibody assay is an *in vitro* diagnostic immunoassay for the qualitative identification of HCV IgG and results are reported in Index Values. Samples with an Index Value less than 0.80 are considered nonreactive, greater than or equal to 0.80 and less than 1.00 are considered equivocal, and greater than or equal to 1.00 are considered reactive. Equivocal samples are repeated in duplicate. If 2 of the 3 sample results are less than 0.80 Index Value, then the sample is considered nonreactive, whereas if 2 of the 3 sample results are greater than or equal to 1.00 Index Value, then the sample is considered reactive and supplemental testing is encouraged. Similarly, if 2 of the 3 sample results are greater than or equal to 0.80 Index Value and less than 1.00 Index Value, then supplemental testing is recommended. HCV antibody was assessed during the transplant work-up period and at unspecified times after transplantation as part of the routine clinical care. Of note, two recipients were noted to seroconvert post-transplant, but upon subsequent testing the HCV antibody was negative. For the purpose of this study, these recipients were treated as positive.

### Data assessment

As described previously [7], HCV RNA and HCV genotype were initially checked between 4 and 8 weeks post-transplant and patients received antiviral therapy shortly thereafter with approved regimens (sofosbuvir/ledipasvir, sofosbuvir/velpatasvir, or glecaprevir/pibrentasvir) for at least 12 weeks. Specifics regarding initiation of DAA therapy are described elsewhere [7]. After the initiation of antiviral treatment, HCV RNA and a comprehensive metabolic panel were checked during treatment at 4, 8, 12 weeks and a final RNA was checked at twelve weeks after antiviral therapy completion.

### Statistical analysis

Baseline data are presented as percentages for categorical variables and as mean ± standard deviation (SD) or median with interquartile range (IQR), as appropriate. Demographic and clinical characteristics associated with HCV seroconversion were assessed using univariate logistic regression modelling. P values were reported as two-sided and defined as statistically significant if <0.05 for all analysis. All analysis was completed using STATA/MP Version 13.1 (STATA Corporation, College Station, TX). The study was approved by the Institutional Review Board of the University of Tennessee Health Science Center (18-06409-XP and 18-06298-XP).

## Results

### Baseline recipient, donor, and transplantation characteristics

We screened 97 transplant recipients who received an HCV antibody positive kidney between 1 March 2018 and 2 December 2019 at our center (Figure 1). Four patients were excluded as they had previous exposure to HCV deemed by a positive HCV antibody prior to transplant. Seven recipients were excluded from this cohort because they received HCV antibody positive, NAT negative donor kidneys. An additional one recipient was excluded as this recipient did not mount evidence of HCV viremia despite receiving HCV antibody positive, NAT positive donor kidney. The final cohort consisted of 85 recipients. Baseline demographic and clinical characteristics of recipients and donors are shown in Table 1. All donors satisfied the criteria of PHS IRO donor. The mean ± SD age of recipients was 53.2 ± 10.8 years, 39% were female, 15% and 84% of patients were white and African American, respectively.

**Table 1.** Baseline and post-transplant characteristics of kidney transplant recipients.

Parameter	Entire cohort	HCV Ab−	HCV Ab+	*p* Value
Observations (n)	85	80	5	
Recipient baseline characteristics				
Age (years), mean (SD)	53.2 (10.8)	53.3 (10.9)	51.6 (9.9)	0.74
Female gender, N, (%)	33 (39%)	32 (40%)	1 (20%)	0.37
Race, N, (%)				
Caucasian	13 (15%)	13 (16%)	0 (0%)	<0.001
Black or African American	71 (84%)	67 (84%)	4 (80%)	
American Indian or Alaska Native	1 (1%)	0 (0%)	1 (20%)	
Ethnicity (Non-Hispanic and Latino), N, (%)	84 (99%)	79 (99%)	5 (100%)	0.80
Insurance (Medicare), N, (%)	71 (84%)	68 (60%)	3 (85%)	0.32
BMI, mean (SD), kg/m^2^	30.0 (5.2)	29.9 (5.3)	31.5 (3.1)	0.51
Recipient Blood Group, N, (%)				
O	31 (36%)	28 (35%)	3 (60%)	0.05
A	39 (46%)	39 (49%)	0 (0%)	
B	6 (7%)	6 (7%)	0 (0%)	
AB	9 (11%)	7 (9%)	2 (40%)	
Dialysis duration (months), median (IQR)	60 (38–82)	60 (37–84)	68 (55–80)	0.75
Cause of End Stage Renal Disease, N, (%)				
Hypertension	41 (48%)	38 (48%)	2 (60%)	0.75
Diabetes	28 (33%)	26 (32%)	2 (40%)	
Glomerulonephritis	12 (14%)	12 (15%)	0 (0%)	
Cystic disease	4 (5%)	4 (5%)	0 (0%)	
Comorbidity, N, (%)				
Diabetes	41 (48%)	38 (48%)	3 (60%)	0.59
Hypertension	84 (99%)	79 (99%)	5 (100%)	0.80
Peripheral vascular disease	11 (13%)	11 (14%)	0 (0%)	0.37
Coronary artery disease	18 (21%)	16 (20%)	2 (50%)	0.29
Chronic obstructive pulmonary disease	4 (5%)	4 (5%)	0 (0%)	0.61
Donor Characteristics				
Age (years), mean (SD)	32.7 (6.5)	32.1 (5.7)	42.4 (10.5)	<0.001
Female gender, N, (%)	37 (44%)	36 (45%)	1 (20%)	0.27
Blood group, N, (%)				
O	31 (36%)	28 (35%)	3 (60%)	0.27
A	43 (51%)	42 (53%)	1 (20%)	
B	6 (7%)	6 (7%)	0 (0%)	
AB	5 (6%)	4 (5%)	1 (20%)	
DCD, N, (%)	8 (9%)	6 (8%)	2 (40%)	0.02
Comorbidity, N, (%)				
Diabetes	1 (1%)	1 (1%)	0 (0%)	0.80
Hypertension	8 (9%)	8 (10%)	0 (0%)	0.46
Peak serum creatinine, mean (SD), mg/dl	1.38 (0.39)	1.38 (0.39)	1.30 (0.28)	0.68
Terminal serum creatinine, mean (SD), mg/dl	0.98 (0.36)	0.98 (0.37)	1.08 (0.37)	0.74
Cause of death, N, (%)				
Anoxia	54 (64%)	50 (62%)	4 (80%)	0.21
Cerebrovascular/Stroke	4 (5%)	3 (4%)	1 (20%)	
Head Trauma	23 (27%)	23 (29%)	0 (0%)	
Other	4 (4%)	4 (5%)	0 (0%)	
Race, N, (%)				
White American	79 (93%)	74 (92%)	5 (100%)	0.94
American Indian or Alaska Native	3 (4%)	3 (4%)	0 (0%)	
Asian American	2 (2%)	2 (3%)	0 (0%)	
Native Hawaiian or Other Pacific Islander	1 (1%)	1 (1%)	0 (0%)	
Ethnicity (Non-Hispanic and Latino), N, (%)	81 (95%)	77 (96%)	4 (80%)	0.10
KDPI, mean (SD)	51 (16)	50 (15)	67 (15)	0.02
Transplant characteristics				
Cold Ischemic Time (min), median (IQR)	1,161 (969–1,390)	1,168 (970–1,397)	1,081 (689–1,325)	0.46
ATG dose (mg/kg), mean (SD)	4.8 (0.8)	4.8 (0.8)	4.6 (0.8)	0.35
HLA A mismatches, N, (%)				
0	4 (5%)	4 (5%)	0 (0%)	0.76
1	24 (28%)	22 (28%)	2 (40%)	
2	57 (67%)	54 (67%)	3 (60%)	
HLA B mismatches, N, (%)				
0	3 (3%)	3 (4%)	0 (0%)	0.49
1	15 (19%)	15 (19%)	0 (0%)	
2	67 (78%)	62 (77%)	5 (100%)	
HLA DR mismatches, N, (%)				
0	11 (13%)	10 (12%)	1 (20%)	0.49
1	39 (46%)	38 (48%)	1 (20%)	
2	35 (41%)	32 (40%)	3 (60%)	
Treatment characteristics				
HCV Genotype, N (%)				
1a	50 (59%)	47 (59%)	3 (60%)	0.93
1b	2 (2%)	2 (2%)	0 (0%)	
2	4 (5%)	4 (5%)	0 (0%)	
3	29 (34%)	27 (34%)	2 (40%)	
Highest level of HCV RNA (IU/ml) before antibody measurement, median (IQR)	290,000 (71,500–952,500)	290,000 (63,700–785,000)	563,000 (127,000–1,620,000)	0.73
HCV Treatment regimen, N, (%)				
GLE/PIB	77 (91%)	73 (91%)	4 (80%)	0.60
SOF/VEL	7 (8%)	6 (8%)	1 (20%)	
SOF/LDV	1 (1%)	1 (1%)	0 (0%)	
HCV RNA level at closest time of antibody measurement (IU/ml), median (IQR)	101,900 (75–417,000)	71,500 (73–313,000)	1,091,500 (345,000–8,360,000)	0.02
Time between transplantation and antibody measurement (days), median (IQR)	210 (180–245)	210 (182–246)	167 (39–213)	0.25
Time between transplantation and viral clearance (days), median (IQR)	237 (223–255)	237 (224–255)	240 (191–260)	0.90

Data was presented as N (%) for categorical variables and mean ± standard deviation (SD) or median and interquartile range (IQR) for continuous variables.

ATG: anti-thymocyte globulin; BMI: body mass index; DCD: donation after cardiac death; GLE: glecaprevir; HCV: hepatitis C virus; HLA: human leukocyte antigen; KDPI: Kidney Donor Profile Index; LDV: ledipasvir; PIB: pibrentasvir; RNA: ribonucleic acid; VEL: velpatasvir.

### HCV seroconversion and its predictors

Of the 85 recipients, 5 (5.9%) recipients were noted to have HCV antibody seroconversion (HCV Ab +) at some point after transplantation. The HCV RNA level at closest time of antibody measurement was higher in the seroconverted patients versus the ones who never converted (median and (interquartile range): 1,091,500 (345,000–8,360,000) vs 71,500 (73–313,000), *p* = 0.02) (Table 1). The groups were otherwise the same except for recipient race (none of the white recipients converted), donor age, Kidney Donor Profile Index (KDPI) and donation after cardiac death (DCD) status (Table 1). Forty percent of the converted recipients versus only 8% of the non-converted recipients received DCD kidney. There was no difference in mean ATG dose or number of human leukocyte antigen mismatches between the group who did not seroconvert (HCV Ab −) and HCV Ab + group.

The emergence of HCV antibody and clinical laboratory characteristics over time are shown in Table 2. A trend in improved creatinine and eGFR was observed after transplantation with treatment of HCV. 

In regard to factors associated with HCV seroconversion, donor DCD status [odds ratio (OR) and 95% confidence interval (CI) was: 8.22 (1.14–59.14)], donor age [OR (95% CI) per 5-year interval was: 3.19 (1.39–7.29)], and KDPI [OR (95% CI) per unit increase was: 1.07 (1.01–1.15)] showed statistically significant association with HCV seroconversion as shown in Table 3. No other clinical or demographic characteristics were associated with HCV seroconversion (Table 3).

**Table 2.** Presence of HCV antibody and liver and kidney markers over time.

All patients	At time of Transplantation	4-weeks after transplantation	8-weeks after transplantation	12-weeks after transplantation	SVR12
Mean Viral Load (IU/mL), median (IQR)	–	516,500 (174,000–1,940,000)	0 (0–110,000)	15 (0–91)	0 (0–0)
Mean ALT (IU/L), median (IQR)	23 (18–29)	41 (29–69)	52 (31–69)	33 (22–47)	29 (22–34)
Mean AST (IU/L), median (IQR)	24 (16–32)	21 (16–39)	29 (20–46)	21 (16–33)	18 (16–22)
Mean creatinine (mg/dL), median (IQR)	8.4 (6.7–10.5)	1.5 (1.2–1.8)	1.4 (1.2–1.7)	1.3 (1.1–1.7)	1.3 (1.1–1.6)
Mean eGFR (ml/min/1.73 m^2^), median (IQR)	7 (5–9)	55 (42–65)	57 (49–69)	60 (50–72)	66 (54–75)
HCV Antibody Present, n (%)	0/0 (0%)	2/12^a^ (17%)	3/16^a^ (19%)	3/19^a^ (16%)	5/85^a^ (6%)

^a^Overlapping recipients.

ALT: alanine aminotransferase; AST: aspartate aminotransferase; eGFR: estimated glomerular filtration rate; HCV: hepatitis C virus; IQR: interquartile range; SVR12: sustained virologic response 12 weeks after the end of treatment.

**Table 3.** Predictors of HCV antibody seroconversion using univariate logistic regression model.

	OR (95% CI)	*p* Value
Recipient age (+1 year)	0.99 (0.91–1.08)	0.73
Recipient gender (female vs male)	0.38 (0.04–3.51)	0.39
Recipient BMI (+1 kg/m^2^)	1.06 (0.90–1.24)	0.51
Recipient time on dialysis (+1 month)	1.00 (0.97–1.03)	0.99
ATG dose (+1 mg/kg)	0.77 (0.25–2.55)	0.70
HCV genotype (3 vs all others)	1.31 (0.27–8.31)	0.77
Donor DCD status (DCD versus non DCD)	8.22 (1.14–59.14)	0.04
Donor age (+5 years)	3.19 (1.39–7.29)	<0.01
KDPI (+1)	1.07 (1.01–1.15)	0.03
HCV RNA level at closest time of antibody measurement (+1,00,000 IU/ml)	1.01 (0.99–1.03)	0.08
Mean AST (+1 IU/L)	0.91 (0.77–1.07)	0.26
Mean ALT (+1 IU/L)	0.96 (0.89–1.04)	0.30

ATG: anti-thymocyte globulin; ALT: alanine aminotransferase; AST: aspartate aminotransferase; BMI: body mass index; KDPI: Kidney Donor Profile Index; HCV: hepatitis C virus; OR: odds ratio; RNA: ribonucleic acid; DCD: donation after cardiac death.

## Discussion

In our study we note the vast majority of kidney transplant recipients receiving HCV-infected organs did not have identifiable HCV antibody despite having HCV viremia. In a recent study conducted under protocolized methods where transplant recipients were immediately treated with DAA after transplantation, Porrett et al. [12] report that 45% of HCV naïve kidney and heart transplant recipients (42% of kidney recipients and 56% of heart recipients) had detectable HCV antibody at some point after transplantation with HCV-infected organs, which is considerably higher than the 6% observed in our cohort. They checked HCV antibody within one week post-transplant in the entire HCV naïve kidney recipient cohort, and interestingly, detection of HCV antibody typically occurred early in the transplant course [12]. One week is most likely an insufficient amount of time for seroconversion in the recipients, as the window period for HCV antibody using chemiluminescence or enzyme immunoassays is ∼40–50 days as compared to a window period of ∼3–5 days for NAT [15]. Indeed, using a novel assay designed to identify the isotype of HCV antibody, the authors note HCV antibody in sera drawn during this first week was IgG, and not IgM, ultimately establishing HCV antibody in HCV naïve recipients occurs *via* donor-derived transmission. Furthermore, the authors discovered HCV antibody persisted beyond 100 days in 4 out of 7 (57%) HCV naïve kidney recipients whom had sera available beyond 30 days post-transplant, leading the authors to determine that HCV antibody is continuously produced in 50% of patients [12]. Similarly, de Vera et al. [13] demonstrated 14 of 32 (44%) HCV naïve kidney transplant recipients receiving HCV antibody positive/NAT negative organs had detectable HCV antibody in the absence of viremia from 1 month to 1 year post-transplant. Taken together, these studies show that passive transfer of donor HCV antibodies occurs in recipients after transplantation of HCV antibody positive organs regardless of NAT status.

Another potential explanation for the difference in percentage of recipients testing positive for HCV antibody in our study when contrasted with other studies [11,12], is that the sensitivity of the test varies depending on reagent and test method utilized [16], subsequently making comparisons challenging.

We did not have specified time-points for routinely checking HCV antibody in our kidney transplant recipients, but the median time between transplantation and antibody measurement was 210  days, which is longer than the ‘window period’ and gives enough time for true seroconversion. However, at 12  weeks post-transplant (∼84 days), 3 out of 19 (16%) HCV naïve kidney transplant recipients had identifiable HCV antibody; at SVR12, 5 out of 85 (6%) HCV naïve kidney transplant recipients had identifiable antibody. Our observation that a small percentage of HCV naïve kidney recipients had detectable HCV antibody runs counter to Porrett et al.’s [12] conclusion that more than half of recipients produce HCV antibody over time in kidney recipients. However in their heart transplant recipients, Porrett et al. [12] reported the antibody level decreased quickly in all patients, dropping below the assay threshold of detection in half of the patients within approximately two weeks of transplant. It remains unclear if the antibody detected at later time points in our study represents passive transmission from donor organs or true seroconversion from viral exposure. Consequently, caution is warranted in interpreting persistent production of HCV antibody given Porrett et al.’s small denominator of 7 HCV naïve kidney transplant recipients.

Interestingly, few donor characteristics [DCD status, age and KDPI (driven by age)] served as predictors of seroconversion in our cohort. In addition, the closest HCV RNA level at antibody measurement was higher in the HCV Ab + group compared to the non-HCV Ab − group. Remarkably, neither time between transplantation and viral clearance, or ATG dose was different between HCV Ab − and HCV Ab + groups. Furthermore, well-powered studies are needed to identify potential predictors of seroconversion this population.

There are several potential explanations of our observations. First, our findings demonstrate the body’s immune response for plasma cells to generate antibodies becomes impaired in the presence of high dose immunosuppression. In particular, depletion of T-cells by ATG might prevent B cell activation and subsequent plasma cell production of HCV antibody. However, similar to our center, Porrett et al. [12] also used ATG as induction treatment. It remains unclear if other forms of induction such as interleukin-2 receptor blockade or high dose corticosteroid induction would produce the same results. Remarkably, two patients had discordant HCV antibody results, initially testing positive but became negative upon repeat antibody evaluation. For example, one patient was noted to have detectable HCV antibody 7 months post-transplant, but upon subsequent testing 8 months after transplant, the HCV antibody was negative. This patient received bortezomib for monoclonal gammopathy of unclear significance. The other patient had an increase in mycophenolic acid dosing between the positive and negative HCV antibody results. de Vera et al. [13] also reported incongruent HCV antibody results at various time-points after transplant despite the absence of viremia. For example, some recipients tested positive at 1-month post-transplant but upon further testing at 3 and 6 months they were noted to be HCV antibody negative; conversely other patients had no HCV antibody detected 1 month after transplant, but were observed to have HCV antibody at 3 and 6 months post-transplantation [13]. Race is often considered as a variable accounting for differences in immunogenicity, with African-Americans often categorized as high-risk particularly for rejection [17]. In our cohort, racial and ethnic minorities, particularly African Americans, American Indian or Alaskan Natives, were more likely to have detectable antibody, whereas no whites had detectable HCV antibody.

While the 2013 US PHS guidelines recommend testing for HIV, HBV, and HCV between 1 and 3 months post-transplant in recipients receiving organs from PHS increased risk donors [4], a retrospective multicenter study demonstrated 40% of patients receiving these do not undergo such screening [5]. In particular, testing for HCV was far from uniform with 25% undergoing screening with antibody alone [5], which is in contrast to the 2013 PHS guideline recommending utilizing HCV NAT to screen for HCV in post-transplant recipients [4]. The AASLD-IDSA provides an option of testing for HCV RNA in those who are immunocompromised [18]. The observation that a considerable majority of our transplant recipients who received HCV-infected kidneys did not have identifiable HCV antibody, indicates HCV antibody is not sufficient to detect active infection in those who are immunosuppressed. This is significant as the updated October 2019 Organ Procurement and Transplantation Network (OPTN) policy calls for transplant programs to develop individual protocols for monitoring transmissible diseases from increased public health risk donors, but little guidance is provided regarding the specific tests to use and timing from the OPTN [19] . As transplantation of HCV-infected organs into HCV naïve recipients becomes more acceptable and streamlined in practice [20], the transplant community’s approach and OPTN’s recommendations should shift to definitively elucidate screening tests and adequate timing. Given the increased utilization of PHS IROs, particularly HCV-infected organs for transplantation in HCV naïve recipients, it may be prudent for HCV screening in transplant recipients to consist of testing HCV RNA as opposed to utilizing HCV antibody. The most recent guideline is consistent with our recommendation [21].

While this study is unique in its findings, it is not without limitations. Data regarding the Index Values of each measurement were not available. Our cohort number is currently the largest reported, but the relatively small number of transplant recipients may result in our study being underpowered to detect true differences. Since this study was done retrospectively, we did not check HCV antibody at pre-specified intervals for all recipients. It is possible some of the 5 transplant recipients who had identifiable HCV antibody did so at an earlier time interval than indicated, as seen in Porrett et al.’s study [12]. However, the absence of HCV antibody in the majority of transplant recipients is notable since the majority of measurements were performed between 6 and 8 months after transplantation, which is outside the window period. Finally, we did not have data on potential confounders such as infection, or non-adherence to immunosuppressive medications, which might contribute to seroconversion these patients.

This work has multiple strengths. To our knowledge, our study evaluated factors associated with HCV antibody seroconversion among the largest cohort of HCV naïve transplant recipients receiving HCV-infected organs. Additionally, we used univariate analysis to assess factors associated with HCV seroconversion. Furthermore, this study represents a ‘real-world’ approach in which transplantation of HCV-infected organs into HCV naïve recipients occurs outside of a pharmaceutical or extramurally sponsored trial and mimics the standard of care in a clinical setting.

## Conclusion

In kidney transplant recipients, HCV antibody should not be used to assess infection, as immunosuppression seems to halt the immune system’s response to establish antibody despite evidence of viremia. Assessing HCV RNA post-transplantation should become routine in transplant recipients receiving PHS IROs.

## Abbreviations

AASLD: American Association for the Study of Liver Diseases
ALT: alanine aminotransferase
AST: aspartate aminotransferase
ATG: anti-thymocyte globulin
DAA: direct antiviral agent
eGFR: estimated glomerular filtration rate
HBV: hepatitis B virus
HCV: hepatitis C virus
HIV: human immunodeficiency virus
IRO: increased risk organ
IDSA: Infectious Diseases Society of America
NAT: nucleic acid testing
OPTN: Organ Procurement and Transplantation Network
PHS: Public Health Service
RNA: ribonucleic acid
SVR: sustained virologic response
